# Quantitative Modeling of Protein Synthesis Using Ribosome Profiling Data

**DOI:** 10.3389/fmolb.2021.688700

**Published:** 2021-06-28

**Authors:** Vandana Yadav, Inayat Ullah Irshad, Hemant Kumar, Ajeet K. Sharma

**Affiliations:** ^1^ Department of Physics, Indian Institute of Technology Madras, Chennai, India; ^2^ Department of Physics, Indian Institute of Technology Jammu, Jammu, India; ^3^ School of Basic Sciences, Indian Institute of Technology Bhubaneswar, Bhubaneswar, India

**Keywords:** ribosome profiling, ribo-seq, translation-initiation rate, codon translation rate, codon usage bias, quantitative modeling of protein synthesis

## Abstract

Quantitative prediction on protein synthesis requires accurate translation initiation and codon translation rates. Ribosome profiling data, which provide steady-state distribution of relative ribosome occupancies along a transcript, can be used to extract these rate parameters. Various methods have been developed in the past few years to measure translation-initiation and codon translation rates from ribosome profiling data. In the review, we provide a detailed analysis of the key methods employed to extract the translation rate parameters from ribosome profiling data. We further discuss how these approaches were used to decipher the role of various structural and sequence-based features of mRNA molecules in the regulation of gene expression. The utilization of these accurate rate parameters in computational modeling of protein synthesis may provide new insights into the kinetic control of the process of gene expression.

## 1 Introduction

Protein molecules carry out a vast array of biological functions. Indeed, almost every cellular process, from genome regulation to energy metabolism, requires a unique set of proteins with their precise concentration in a cell (Berg et al., 2002; Miyazaki and Esser, 2009). Therefore, the abundance of proteins in a cell is tightly regulated by various mechanisms acting at the level of transcription and translation (Curtis et al., 1995; Wu and Belasco, 2008). Understanding this regulation of protein synthesis remains one of the active areas of research from the last few decades (Merrick, 1992; Proud, 2006; Dever et al., 2016; Kummer and Ban, 2021). It was previously believed that cellular protein levels are primarily determined by mRNA copy number (Greenbaum et al., 2003; Lu et al., 2007). However, recent studies demonstrated that the translational regulation of protein synthesis also contributes significantly to *in vivo* protein abundance (Vogel et al., 2010; Li et al., 2014). Translational regulation is achieved by various structural and sequence-based features of mRNA molecules that can kinetically control the rate of protein synthesis (Kudla et al., 2009; Vogel et al., 2010; Tuller et al., 2010). Therefore, the knowledge of the rates at which different steps of translation occur and their connection with those mRNA features would provide key information concerning how the expression of an individual gene is regulated.

Single-molecule experiments provided significant insights into the details of the process of protein synthesis (Munro et al., 2008; Volkov and Johansson, 2018). However, little was known on how different mRNA features can control protein synthesis until the Next Generation Sequencing (NGS) experiments started uncovering various kinetic properties of this process (Ingolia et al., 2019). Several methods have been developed in the last decade that allows the accurate extraction of translation rate parameters using ribosome profiling data (Weinberg et al., 2016; Duc and Song, 2018; Sharma et al., 2019). The analysis of these translation rate parameters and their use in protein synthesis simulations have started unraveling the sophisticated mechanism that nature has developed to optimize the use of cellular resources (Tuller et al., 2010; Diament et al., 2018; Lyu et al., 2020).

In this mini-review, we provide the brief overview of a few recently developed methods that extract translation rate parameters from ribosome profiling data. We explain the technical details and assumptions made in these approaches, and also comment on their accuracy in several different contexts. We also highlight some of the recent results that use ribosome profiling data to provide new insights into the kinetic control of protein synthesis. This mini-review aims to promote the use of big biological data sets among biophysicists, biophysical chemists and system biologist to achieve greater accuracy and reliability in the quantitative modeling of protein synthesis.

## 2 Quantitative Modeling of Protein Synthesis

**FIGURE 1:**
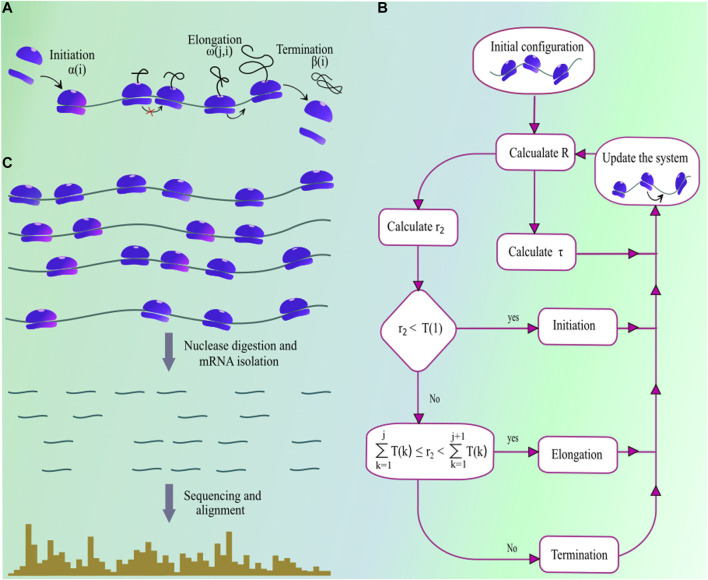
**(A)** A pictorial illustration of the steps involved in the process of protein synthesis. Ribosome subunits assemble at the start codon with rate α(i) when no ribosome occupies the first six codons of the transcript. Then, the ribosome starts moving toward the stop codon by a single codon at a time. It hops from codon *j* to j+1 with rate ω(j,i). Note well, a ribosome cannot move to the next codon if it is occupied by another ribosome. After arriving at the stop codon, the ribosome terminates protein synthesis and releases fully synthesized protein with rate β(i) **(B)** The flow-chart describes the computational algorithm of protein synthesis simulations using the Gillespie’s method. For details, see the main text and table (1) **(C)** explains various experimental and computational steps involved in preparing ribosome profiles.

The first mathematical model of protein synthesis was developed by MacDonald et al. (1968), and since then various similar models and their extensions were proposed to understand the different aspects of mRNA translation (Garai et al., 2009; Sharma and Chowdhury, 2012; Ciandrini et al., 2013; Margaliot and Tuller, 2013). Among these, the totally asymmetric simple exclusion process (TASEP) is a model which incorporates key steps regulating *in vivo* protein production and is extensively used to stimulate protein synthesis. In this model, an mRNA is considered as a one-dimensional lattice where each site in that lattice represents a single codon. A ribosome in this model is like an extended particle that covers ten consecutive codon positions of a transcript where its location is usually identified by the position of its A-site (Sharma and O’Brien, 2017; Ingolia et al., 2009). The ribosome A-site is located at the sixth codon from the 5′ end of the ribosome. Therefore, a ribosome at the $j^{th}$ position covers j−5 to j+4 codons (Ahmed et al., 2019). Protein synthesis in this model is divided into three sub-steps: initiation, elongation and termination (Merrick, 1992) (Figure 1A). Initiation occurs when a ribosome assembles at the start codon of the $i^{th}$ transcript with rate α(i) (Kozak, 1999; Merrick and Pavitt, 2018). A ribosome initiates protein synthesis with its A-site at the second codon position of the transcript, therefore the translation initiation occurs when the first six codons are not occupied by another ribosome. The limited availability of ribosomes in a cell makes translation-initiation a rate-limiting step of the protein synthesis (Shah et al., 2013). The ribosome then starts taking a series of stochastic steps toward the stop codon. On the $i^{th}$ transcript, a ribosome slides from codon position *j* to j+1 with rate ω(j,i) **.** In each of such steps, a ribosome selects cognate aa-tRNA molecule, forms a peptide bond and then moves to the next codon position **(** Sharma and Chowdhury, 2011; Sharma and Chowdhury, 2012 **)** Note that multiple ribosomes simultaneously move on a single transcript where each of them synthesizes a separate copy of protein. Therefore, due to mutual exclusion, a ribosome cannot move to the next codon if its passage is blocked by another downstream ribosome. After arriving at the stop codon, the ribosome terminates the process, and releases a fully synthesize protein with rate β(i). The termination of protein synthesis also leads to the disassembly of the ribosome at the stop codon (Hellen, 2018).

The TASEP with an uniform elongation rate, unitary particle size, and infinite lattice size is exactly solved by Derrida et al. (1992). Later, Lazarescu and Mallick (2011) extended this work by solving the TASEP with finite lattice size. Adding to this, Kolomeisky (1998) provided an analytical solution for TASEP with local inhomogeneities. For extended particles, Shaw et al. (2004) solved the TASEP model under mean-field approximation for uniform elongation rate. Recently, Szavits-Nossan et al. (2018) provided an analytical solution for the TASEP with non-uniform elongation rate and extended particle size; however, it ignores the higher-order terms of power series solution of the model. Therefore, in the absence of any exact analytical solution, computer simulation of the TASEP model is the best possible approach for making reliable quantitative predictions on protein synthesis.

Multiple approaches have been used in the past to simulate protein synthtesis on TASEP model, including kinetic Monte-Carlo (Zia et al., 2011), next reaction method (Gibson and Bruck, 2000; Duc and Song, 2018), Gillespie’s method (Gillespie, 1977), etc. The Gillespie’s method is one of the very efficient approach for studying stochastic systems and is commonly used to simulate the TASEP model (Gillespie, 1977). Solving TASEP using this method requires to calculate the parameter

$$R=\overset{L_{i}}{\underset{j=1}{\sum }}T\left(j\right)$$ (1)

which is the sum total of rates for all transitions that lead to a new state from the current state of the translation system. (Note that every unique arrangement of ribosomes on a transcript is a separate state.) In Eq. 1, T(1)=α(i), T(j)=ω(j,i)δ(j) for $2\leq j<L_{i}-1$ and $T\left(L_{i}\right)=\beta \left(i\right)\delta \left(L_{i}\right)$; δ(j) is one when a ribosome occupies the $j^{th}$ codon position of the transcript, otherwise it is zero, and $L_{i}$ is the total number of codons in the $i^{th}$ transcript. The TASEP model assumes that all transitions in translation system are Markovian therefore, the dwell time in a given state is exponentially distributed with a mean value of $\frac{1}{R}$. This quantity is calculated by generating an exponentially distributed random number $\tau =\frac{1}{R}ln\left(r_{1}\right)$, where $r_{1}$ is a random number that is uniformly distributed between 0 and 1. The next transition is randomly chosen according to the relative contributions of all possible transitions in *R* (Eq. 1). For this, another random number $r_{2}$, which is uniformly distributed between 0 and *R*, is generated, and the next transition is identified according to the selection criteria given in Table 1 (A flow-chart explaining the various steps of protein synthesis simulations is shown in Figure 1B). Repeating this procedure generates a trajectory of protein synthesis on a given mRNA transcript, which can be used to calculate various quantities: rate of protein synthesis, average and codon-specific ribosome density, in-silico ribosome profiles, etc.

**TABLE 1:** A list of the types of transitions and conditions in simulation algorithm.

Conditions	Transitions
$r_{2}<T\left(1\right)$, and the first six codon positions of the transcripts are unoccupied	Translation-initiation
$\overset{j}{\underset{k=1}{\sum }}T\left(k\right)\leq r_{2}<\overset{j+1}{\underset{k=1}{\sum }}T\left(k\right)$, a ribosome is at the $j^{th}$ codon position with no ribosome at (j+10)	Transition of the ribosome from codon position *j* to j+1
$\overset{L_{i}-1}{\underset{k=1}{\sum }}T\left(k\right)\leq r_{2}<\overset{L_{i}}{\underset{k=1}{\sum }}T\left(k\right)$, a ribosome is at the $L_{i}^{th}$ codon	Translation-termination

## 3 Methods of Extracting Translation Rate Parameters

Accurate quantitative predictions for protein production critically depends on having precise estimates of translational rate parameters. Therefore, in this section, we discuss common approaches to extract translation rate parameters from ribosome profiles which provides the access to a plethora of information about the protein synthesis process. In ribosome profiling experiment, translation elongation is arrested by the treatment with drug cycloheximide (Figure 1C). Then, after the cell lysis, the portions of the mRNA molecule that are not protected by ribosomes are digested by nucleases. The remaining ribosome-protected mRNA fragments are subsequently sequenced and aligned to a reference genome. The typical length of a ribosome-protected mRNA fragment is 28–31 nucleotides; therefore, fragments outside this range are excluded from the analysis (Pop et al., 2014). Note that a ribosome translates the codon present at its A-site. Therefore, the position of A-site is identified on those ribosome-protected mRNA fragments (Ahmed et al., 2019), referred to as the read aligned to the A-site codon. Within a transcript, more ribosome-reads aligned to a codon means a longer translation time for that particular codon. This steady-state profile of ribosome occupancy can be used in extracting translation initiation and codon translation rates. Many computational methods have been developed in the past to extract these rate parameters from ribosome profiling data. We categorize these methods into three different groups.

### 3.1 Optimization Based Methods

These methods take advantage of some known characteristics of the translation process and extract codon translation rates by optimizing or fitting a function of ribosome profiling reads. For example, Dana and Tuller (2014) proposed a method for extracting the “typical” translation time of a codon by fitting a function that characterizes ribosome dwell time distribution. To do that, Dana and Tuller (2014) first normalize the ribosome footprint counts by the average reads aligned to the transcript. That is,

$$\hat{r}\left(j,i\right)=\frac{r\left(j,i\right)}{\frac{1}{L_{i}-40}\sum_{j=21}^{L_{i}-20}r\left(j,i\right)}$$ (2)

r(j,i) in Eq. 2 is the number of ribosome profiling reads aligned to the $j^{th}$ codon of the $i^{th}$ transcript. To minimize any sampling error, the authors recommend excluding the genes with median read counts less than one. In addition to that, the first and the last 20 codons were removed from the analysis as unusually high ribosome footprint density was found in those regions (Ingolia et al., 2009). Then, the distribution of normalized footprint count for a codon type “*c*”(*e.g.*, CUU, AUG) is computed using the data collected from the whole transcriptome. It was found that this distribution is a superposition of the normal and exponential distributions (Dana and Tuller 2014; Dana and Tuller , 2015). Authors rationalize this observation by proposing that the normal distribution characterizes the typical decoding time for a codon type ($\tau_{1}$) whereas the exponential distribution reflects the delay ($\tau_{2}$) caused by rare translation pauses and ribosomal interference. Therefore, the distribution of codon translation time (*τ*), a sum of random variables $\tau_{1}$ and $\tau_{2}$, is the convolution of the normal and exponential distribution, and has the following functional form.

$$f\left(\tau ,\mu_{c},\sigma_{c},\lambda_{c}\right)=\frac{\lambda_{c}}{2}e^{\frac{\lambda_{c}}{2}\left(2\mu_{c}+\lambda_{c}\sigma_{c}^{2}-2\tau \right)}\text{erf}\left[\frac{\mu_{c}+\lambda_{c}\sigma_{c}^{2}-\tau }{\sqrt{2}\sigma_{c}}\right]$$ (3)

In Eq. 3, $\mu_{c}$ and $\sigma_{c}$ are the mean and standard deviation of the normal distribution, respectively. $\lambda_{c}$ is the coefficient for exponential distribution whose inverse is the average time delay caused by rare translation pauses and ribosomal interference. The distribution in Eq. 3 was then fitted with normalized read distribution of codon “*c*”, providing $\mu_{c}$, the “typical” codon translation time. Using this analysis, the authors calculate the ‘typical’ decoding time for all 61 sense codons.

Pop et al. (2014) proposed a similar method that calculates gene-specific and globally averaged translation time of a codon type “*c*” (*i.e.*, $\mu_{c}^{i}$ and $\mu_{c}$, respectively) by maximizing the following objective function.

$$max\left(\mu_{c}^{i},\mu_{c}\right)\left[log\;\underset{i,j}{\prod }\mu_{c}^{i\left(r\left(j,i\right)/J_{i}\right)}e^{-\mu_{c}^{i}}-\underset{i,c}{\sum }w_{c}^{i}{\left(log\mu_{c}^{i}-log\mu_{c}\right)}^{2}\right]$$ (4)

The first term in the objective function represents the likelihood of observed ribosome profiles for a specific $\mu_{c}^{i}$ whereas the second term minimizes the difference between the global and gene-specific average translation time. $w_{c}^{i}$ in Eq. 4 is a ratio of the total number of “*c*” codons in the $i^{th}$ transcript to all transcripts. Therefore, the genes with more “*c*” codons will have greater weightage in the objective function. $J_{i}$ is the ribosome flux at transcript *i* which was fixed to $\underset{j\in \;i\;}{\sum }\frac{r\left(j,i\right)}{L_{i}}$. Then, L-BFGS algorithm (Byrd et al., 1995) is applied to search the numerical values of $\mu_{c}^{i}$ and $\mu_{c}$ that maximizes the objective function in Eq. 4. The inverse of the $\mu_{c}$ is the translation rate of codon “*c*”.

In another optimization based approach, Szavits-Nossan and Ciandrini (2020) have used the non equilibrium analysis of ribosome profiling data to infer the ratio of elongation to initiation rate *i.e.* $k\left(j,i\right)=\frac{\omega \left(j,i\right)}{\alpha \left(i\right)}$. In this method, k(j,i)s were calculated by minimizing the difference between experimentally measured and numerically computed codon-specific ribosome density of a transcript. For this, an objective function

$$S=\overset{L_{i}}{\underset{j=2}{\sum }}{\left(\rho^{ana}\left\{k\left(j,i\right)\right\}-\rho \left(j,i\right)\right)}^{2}$$ (5)

is minimised by using the Least-Squared optimization technique. $\rho^{ana}\{k(j,i)\}$ in Eq. 5 is numerically computed by using an expression derived by Szavits-Nossan et al. (2018) whereas ρ(j,i)s were calculated by distributing the experimentally measured polysome density according to the distribution of ribosome profiling reads along a transcript. Optimizing the objective function in Eq. 5 yields the numerical values of k(j,i). Unlike Pop et al. (2014) and Dana and Tuller (2014), this method does not put any constraint on the variations in the translation rate of a codon type. Therefore, the normalized rates obtained from this method can precisely capture the local variation in codon translation rates along a transcript.

### 3.2 Simulation Based Methods

Simulation based methods follow an iterative procedure, where in each iteration, translation rate parameters are altered until the simulation output converges to its experimentally measured counterparts. For example, Gritsenko et al. (2015) compared the experimentally measured and simulated ribosome densities on mRNA segments of different lengths. This comparison on short mRNA segments allows capturing the local variation in codon translation rates whereas in long segments it increase the reliability of measured rate parameters. These mRNA segments were constructed by dividing the whole transcript into two parts in such a way that both of them get an equal number of combined ribosome profiling and RNA-seq reads. The daughter segments were further divided recursively using the same approach until a reliable estimate of ribosome density can be made.

To implement this method, Gritsenko et al. (2015) used an experimental observation that ribosome density on a mRNA segment is distributed log-normally among all its replicates. This means the probability of finding a specific value of ribosome density in a single observation can be expressed as

$$P\left(C,N_{k}^{i}|r_{k}^{i},\sigma_{k}^{i}\right)\propto \frac{1}{N_{k}^{i}\sqrt{2\pi }}e^{\frac{lnN_{k}^{i}+lnC-r_{k}^{i}}{2{\left(\sigma_{k}^{i}\right)}^{2}}}$$ (6)

$r_{k}^{i}$ and $\sigma_{k}^{i}$ in Eq. 6 are the mean and standard deviation of ribosome density for the $k^{th}$ segment of transcript *i*; $N_{k}^{i}$ is the number of ribosomes on the same mRNA segment which was observed in a single snapshot of protein synthesis simulations. The experimental ribosome density was calculated by taking the ratio of the normalized ribosome profiling reads with the RNA-Seq reads aligned to the same segment. Since this quantity is measured in arbitrary units, a parameter *C* was introduced to scale it to the simulated ribosome density. $r_{k}^{i}$ in Eq. 6 was calculated by taking the mean of ribosome density from the data collected from all replicates of the same experiment. The shape parameter $\sigma_{k}^{i}$ was calculated for a group of segments with the same length as it was not possible to reliably calculate $\sigma_{k}^{i}$ from a very small number of replicates. Then, using Eq. 6, Gritsenko et al. (2015) define an objective function Ψ that quantifies how well the simulation model predicts experimentally measured ribosome densities.

$$\text{Ψ}=\underset{i}{\sum }\underset{k}{\sum }\left[-\frac{1}{2\sigma_{k}^{i}}{\left(lnN_{k}^{i}-r_{k}^{i}+lnC\right)}^{2}-lnN_{k}^{i}\right]$$ (7)

Gritsenko et al. (2015) carry out the transcriptome-wide protein synthesis simulations by supplying some initial translation rate parameters to the model. Snapshots taken from protein synthesis simulations produced $N_{k}^{i}$ for each mRNA segment which were used to compute Ψ in the model evaluation step. Then, using the numerical value of Ψ, a genetic algorithm (Covariance Matrix Adaptation Evolutionary Strategy) proposes new initiation and codon translation rates, which were further used to simulate protein synthesis on the whole transcriptome. This process is repeated until Ψ is maximized which produces the translation initiation and codon translation rates.

In an another simulation based study, Duc and Song (2018) measured translation rate parameters by comparing the normalized *in vivo* ribosome profiling reads (Eq. 2) with those obtained from simulations. This method also requires initial translation rate parameters to generate in-silico ribosome profiles which will be refined in every iteration of the method. Initial codon translation rates were estimated as follows.

$$\omega \left(j,i\right)=\{\begin{array}{cc} min\left(\omega_{max},\frac{\sum_{j=1}^{L_{i}}r\left(j,i\right)}{r\left(j,i\right)}\right), & \text{if }r\left(j,i\right)\neq 0\\ \omega_{max}, & \text{otherwise} \end{array}$$ (8)

$\omega_{max}$ here is a crude guess of the maximum codon translation rate in *S. cerevisiae*. The initial translation initiation rates were calculated by following a method developed by Ciandrini et al. (2013). In that method, initiation rate of a transcript is varied until the simulated average ribosome density matches with what has been measured in polysome profiling experiments. These initial translation rate parameters were then used to simulate protein synthesis and generate *in silico* ribosome profiles. In each step, simulated and *in vivo* ribosome profiles were compared and error *ε* is computed for each codon of a transcript.

$$\varepsilon \left(j\right)=\left|\frac{r\left(j,i\right)}{\sum_{j}r\left(j,i\right)}-\frac{r^{sim}\left(j,i\right)}{\sum_{j}r^{sim}\left(j,i\right)}\right|$$ (9)

Authors also defined codon positions with significant error where it was larger than $\frac{10\sum_{j}\varepsilon \left(j\right)}{L_{i}}$. It is very likely that ribosome profiling reads at such positions may have been influenced by extensive ribosome traffic-jams which increases the dwell time of downstream ribosomes. Therefore, for such cases, translation rate of neighboring codons were also updated in the next iteration cycle. Authors used the following updating rules in each iteration of the method.

$$\omega \left(j,i\right)=\{\begin{array}{cc} \lambda_{1}\omega \left(j,i\right) & \text{for codon }j\text{ where }\varepsilon \left(j\right)\;<\;\frac{10\sum_{j}\varepsilon \left(j\right)}{L_{i}}\\ \lambda_{2}\omega \left(j,i\right), & \text{for codons }j-30\text{ to }j-10\text{ when }\varepsilon \left(j\right)\;>\;\frac{10\sum_{j}\varepsilon \left(j\right)}{L_{i}}\\ \omega \left(j,i\right) & \text{otherwise} \end{array}$$ (10)

$\lambda_{1}$ and $\lambda_{2}$ are chosen using the golden section search algorithm. After updating the codon translation rates, a transcriptome-wide simulations of protein synthesis were carried out and then the same procedure is repeated until no error sites were detected. This provides the translation initiation and codon translation rates that generates *in silico* ribosome profiles similar to the *in vivo* profiles.

### 3.3 Chemical Kinetic Based Methods

Chemical kinetic-based methods do not require extensive simulations of protein synthesis. Instead, they rely on analytical expressions of translation rate parameters that use ribosome profiling and RNA-Seq data as input variables. A recent publication used the mean-field and steady-state assumptions, and derived the following analytical expressions for translation-initiation and codon translation rates (Sharma et al., 2019).

$$\alpha \left(i\right)=\frac{\langle \rho \left(i\right)\rangle \left(L_{i}-1\right)}{\langle TT\left(i\right)\rangle \left[1-\sum_{k=2}^{\mathrm{11}}\rho \left(k,i\right)\right]}$$ (11)

$$\omega \left(j,i\right)=\frac{\sum_{j=2}^{L_{i}-1}\rho \left(j,i\right)}{\rho \left(j,i\right)\langle TT\left(i\right)\rangle }$$ (12)

In Eq. 11, Eq. 12 〈TT(i)〉 is the time a ribosome takes to move from the start to stop codon whereas ρ(i) is the average ribosome density on the transcript. 〈TT(i)〉 can be calculated by using a scaling relation between gene translation time and the number of codons in a transcript (Sharma et al., 2018) if gene-specific reliable estimates of protein synthesis times are not available. The average ribosome density on a transcript is proportional to the ratio of the number of ribosome profiling and RNA-Seq reads aligned to that transcript. This proportionality was used to estimate the average ribosome density on a transcript (Sharma et al., 2019). ρ(j,i)s were calculated by distributing the ρ(i)s according to the variations in ribosome profiling reads across the transcript *i*. This new analysis method neither relies on heuristic and ad-hoc approaches nor requires extensive protein synthesis simulations, and implementing this equation-based method is much easier than others.

## 4 Statistical Noise and Sequence Biases in Measured Translation Rate Parameters

Ribosome profiles provide single codon resolution to the protein synthesis process. However, these data sets are very noisy and are subjected to numerous biases associated with various steps of the ribosome profiling experiment, including the amplification of ribosome footprints by RT-PCR, nuclease digestion, cell lysis, etc. (O’Connor et al., 2016; Mohammad et al., 2019; Xiao et al., 2016; Hussmann et al., 2015 **)**. Such statistical errors and biases will also be reflected in the measured translation rate parameters. Dana and Tuller (2014) minimize their impact by ignoring any variation in the translation rate of a codon type. This approach drastically reduces the total number of parameters to be extracted from the ribosome profiling data. It minimizes the statistical uncertainty in the measurement of codon translation rates and also averages out various sequence biases. However, a major drawback of this approach is that it does not account for the context-dependent variations in codon translation rates. The other extreme approach taken by Szavits-Nossan and Ciandrini (2020) measures the translation rate for each codon in an mRNA transcript but such measurements are subjected to a higher degree of stochastic noise. A few probabilistic and machine learning models have also been applied to minimize the effect of noise and biases in the identification of A-site position on ribosome footprints (Fang et al., 2018; Tunney et al., 2018; Gobet et al., 2020). These models have successfully captured the context-dependent variation in codon translation rates and also performed well in transcripts with low abundance (Liu et al., 2020; Michel et al., 2016).

## 5 Molecular Determinants of Translation Rate Parameters

A closer look at the measured translation rate parameters unraveled the molecular determinants of translation-initiation and codon translation rates (Sharma et al., 2019; Duc et al., 2018). For example, tRNA pool hypothesis proposed codon translation rates to be proportional to the availability of cognate tRNA molecules (Ikemura, 1981; Ikemura, 1985). However, this hypothesis was never explicitly tested as there was no method that allowed translation rate measurement for all codons. Codon translation rates measured by ribosome profiling experiments supported this hypothesis in *S. cerevisiae* and *E. coli* (Dana and Tuller, 2014; Sharma et al., 2019). However, no such behaviour was observed in mouse cell lines (Gobet et al., 2020; Ingolia et al., 2011). Interestingly, a strong correlation between codon usage and tRNA abundance is observed in mammalian cells (Gobet et al., 2020; Gobet and Naef, 2017; Neelagandan et al., 2020), suggesting that tRNA levels are tuned according to their requirement in a cell.

**FIGURE 2:**
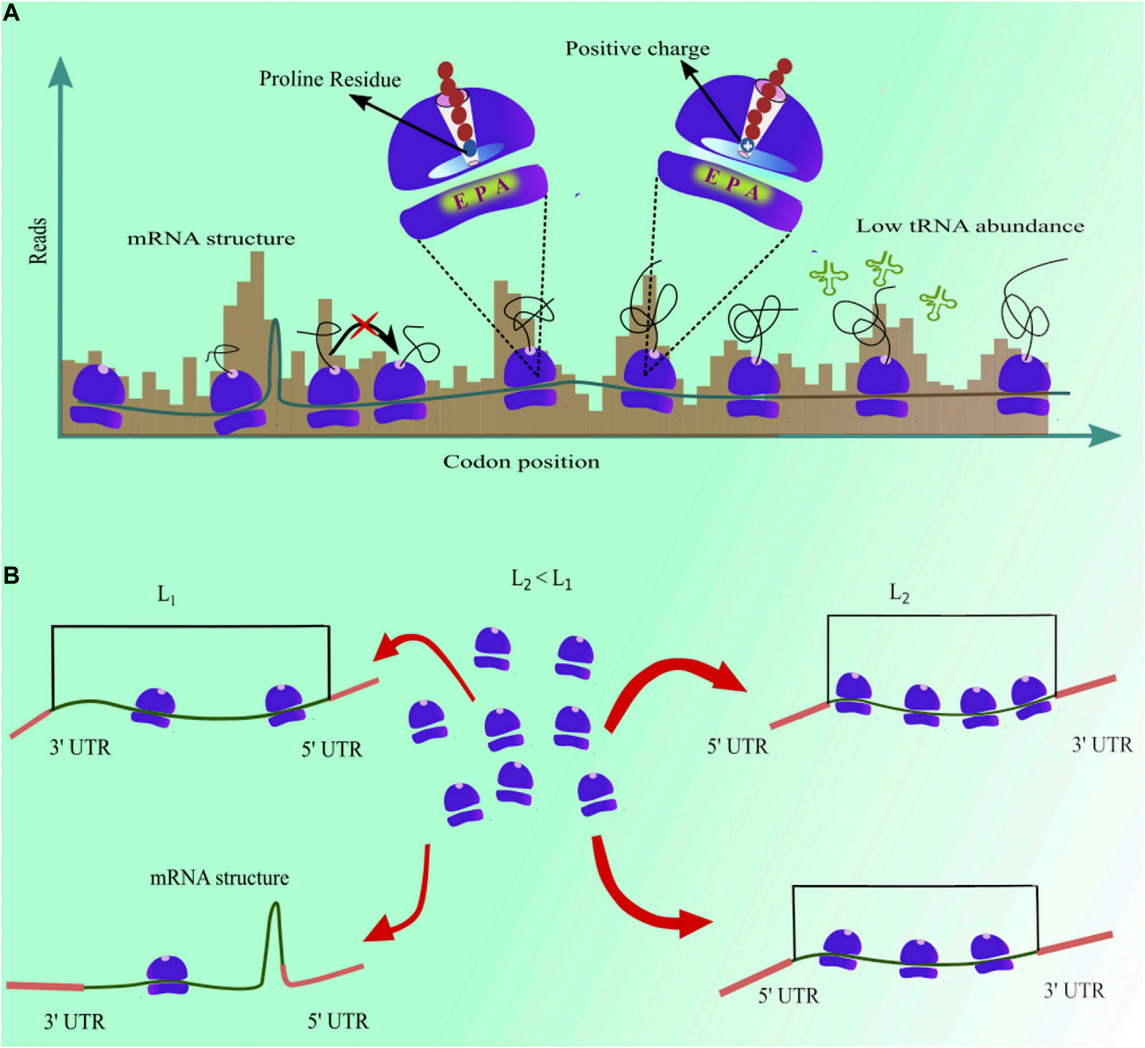
**(A)** mRNA structure, low tRNA abundance, and the presence of proline and positively charged amino acids in ribosome exit tunnel increase ribosome dwell time, resulting in higher ribosome profiling reads for those codon positions **(B)** Presence of mRNA structure at the 5′ end and length of the coding sequence in a transcript anti-correlate with initiation rate.

In addition, an analysis of measured codon translation rate has shown that mRNA structures downstream to the A-site codon increase their translation time (Sharma et al., 2019). The reason for this increase is that the ribosome has to first unfold the structure to proceed to the next codon (Qu et al., 2011). The average increase of 6.7% in codon translation time was reported due to the presence of a structure in mRNA molecule. However, depending upon the stability of that structure, it may vary from one codon to another. Similarly, the presence of proline amino acid on the ribosome P-site increases the median translation time of a codon by 19% (Sharma et al., 2019) because the stereochemistry of proline amino acid delays the peptide bond formation with adjacent amino acid (Pavlov et al., 2009). Furthermore, Duc and Song (2018) have discovered that the aqueous environment inside the ribosome exit tunnel leads to a faster translation of codons when hydrophobic amino acid residues are present inside the tunnel. Electrostatic charges on the nascent-protein have been shown to modulate the translation elongation rate (Duc and Song, 2018; Riba et al., 2019). The ribosome exit tunnel is negatively charged. Therefore, the presence of positively charged amino acid into the tunnel decreases the translation rate of downstream codons (Figure 2A). The identity of the amino acid at the ribosome P-site also affects the translation rate of the A-site codon. In *S. cerevisiae*, eighty six different pairs of amino acids at the A- and P-sites speed up the elongation rate whereas it is slowed down in the case of eighty one other pairs of amino acids (Ahmed et al., 2020). A similar behaviour was also observed in mouse liver cells where different codon combinations at A- and P-sites, E- and P-sites, and E- and A-sites can speed up or slow down the translation elongation (Gobet et al., 2020). Post-translational modifications of tRNA molecules also enhances the rate of translation elongation in *S. cerevisiae*, *N. crassa* and *C. elegans* (Lyu et al., 2020; Nedialkova and Leidel, 2015). In addition to these molecular factors, patterns of slow and fast codons can cause ribosome traffic-jams on a transcript, and can significantly affect the time a ribosome spends at a given codon position (Diament et al., 2018; Duc et al., 2018).

Translation initiation is another rate-limiting step (Shah et al., 2013) and sets an upper bound to the rate at which proteins are produced from a single transcript (Szavits-Nossan and Ciandrini, 2020). Initiation rates measured from ribosome profiling experiments also identified its molecular determinants (Figure 2B). For example, significant negative correlations of initiation rate with the free energy of mRNA folding near the start codon and transcript length were observed in multiple studies (Weinberg et al., 2016; Duc et al., 2018; Sharma et al., 2019). A stable structure near the start codon makes this region inaccessible to a ribosome for initiating the process of protein synthesis, thus decreasing the translation initiation rate. Indeed, many mRNA sequence design algorithms minimize folding energy in this region to enhance the production heterologous proteins (Salis et al., 2009; Angov, 2011). Similarly, the length of the coding sequence of a transcript is inversely proportional to the translation initiation rate (Duc and Song, 2018; Sharma et al., 2019). It is because the ribosomes completing protein synthesis at the termination end can easily diffuse to the start codon in shorter mRNA transcripts (Fernandes et al., 2017). These faster initiation rates in shorter transcripts help them in producing more proteins. Moreover, the presence of AUG codons upstream to the start codon can interfere in the recruitment of ribosomes for translation initiation, thus resulting in a decrease in the translation initiation rate (Sharma et al., 2019). Furthermore, the presence of KOZAK sequence in *S. cerevisiae* transcripts leads to a faster initiation as it serves as a stable binding site for the small ribosome subunit (Sharma et al., 2019). These new findings have demonstrated that a combination of several molecular factors work in tandem to finely modulate the translation-intiation and codon translation rates.

## 6 Concluding Remarks and Future Directions

The development of ribosome profiling has allowed access to relative ribosome occupancy at single codon resolution (Ingolia et al., 2009; Ingolia et al., 2019). Many computational tools can convert this time-independent steady-state information into the kinetic rate parameters of protein synthesis (Dana and Tuller 2014; Pop et al., 2014; Szavits-Nossan and Ciandrini, 2020). Analysis of these rate parameters and their use in protein synthesis simulations give significant insight into the translational regulation of an individual gene (Shah et al., 2013; Lyu et al., 2020). These rate parameters also help identify various structural and sequence-based mRNA features that control the rate of protein synthesis (Weinberg et al., 2016; Duc et al., 2018; Sharma et al., 2019). Furthermore, the knowledge of these rates offers an unprecedented opportunity to explore and model other parallel and downstream processes influenced by translation-elongation kinetics, including co-translational protein folding, mRNA degradation, protein translocation through a membrane, chaperone binding, post-translational modifications, etc (Sharma and O’Brien, 2018; Radhakrishnan et al., 2016).

Analysis of measured translation rate parameters demonstrated that a combination of multiple molecular factors determines the translation-initiation and codon translation rates (Sharma et al., 2019). The strength with which these molecular factors act on translation rate parameters can vary from one place to another. Therefore, the same codon at two different locations can be translated at different rates. Current approaches can capture this context-dependent variation in codon translation rates (Sharma et al., 2019; Szavits-Nossan et al., 2018). However, none of them can quantify the impact of each molecular factor on translation-initiation and codon translation rates. Decoupling their effects would enable the scientists to make reliable predictions on translation rate parameters by only looking at mRNA sequence features, thus providing deeper insights into the context-dependent variation in codon translation rates.

Reliable predictions on protein synthesis, from a single transcript to the whole-cell level, are required for numerous synthetic biology applications (Purcell et al., 2013; Burke et al., 2020). For example, carefully placing the molecular determinants of translation rate parameters may help in designing heterologous genes and synthetic biological circuits. Moreover, in the absence of reliable gene expression models, synthetic biology relies heavily on the trial and error approach (Purcell et al., 2013; El Karoui et al., 2019). Therefore, the accurate predictions made by the quantitative models of protein synthesis will speed up the whole process of designing synthetic biology products with potential applications in areas such as drug delivery, cellular engineering, next-generation drugs, deployable medical devices, etc (Salis et al., 2009; Goldberg et al., 2018; Macklin et al., 2014).
